# Empagliflozin lessened cardiac injury and reduced visceral adipocyte hypertrophy in prediabetic rats with metabolic syndrome

**DOI:** 10.1186/s12933-016-0473-7

**Published:** 2016-11-11

**Authors:** Hiroaki Kusaka, Nobutaka Koibuchi, Yu Hasegawa, Hisao Ogawa, Shokei Kim-Mitsuyama

**Affiliations:** 1 Department of Pharmacology and Molecular Therapeutics, Kumamoto University Graduate School of Medical Sciences, 1-1-1 Honjo, Kumamoto, 860-8556 Japan; 2 Department of Cardiovascular Medicine, Kumamoto University Graduate School of Medical Sciences, Kumamoto, Japan

**Keywords:** Cardiac protection, Oxidative stress, Inflammation, Metabolic syndrome, Prediabetes, Natriuresis, Adipose tissue

## Abstract

**Background:**

The potential benefit of SGLT2 inhibitors in metabolic syndrome is with prediabetic stage unclear. This work was undertaken to investigate the non-glycemic effect of empagliflozin on metabolic syndrome rats with prediabetes.

**Methods:**

SHR/NDmcr-cp(+/+) rats (SHRcp), a model of metabolic syndrome with prediabetes, were given empagliflozin for 10 weeks to examine the effects on urinary sodium and water balance, visceral and subcutaneous adipocyte, and cardiac injury. Further, the effect of empagliflozin on blood pressure and autonomic nervous system was continuously investigated by using radiotelemetry system.

**Results:**

Empagliflozin significantly reduced urinary sodium and water balance of SHRcp only within 1 week of the treatment, but later than 1 week did not alter them throughout the treatment. Empagliflozin significantly reduced body weight of SHRcp, which was mainly attributed to the significant reduction of subcutaneous fat mass. Empagliflozin significantly reduced the size of visceral adipocytes and increased the number of smaller size of adipocytes, which was associated with the attenuation of oxidative stress. Empagliflozin ameliorated cardiac hypertrophy and fibrosis of SHRcp, in association with the attenuation of cardiac oxidative stress and inflammation. However, empagliflozin did not significantly change blood pressure, heart rate, sympathetic activity, or baroreceptor function, as evidenced by radiotelemetry analysis.

**Conclusions:**

Our present work provided the evidence that SGLT2 inhibition reduced visceral adipocytes hypertrophy and ameliorated cardiac injury in prediabetic metabolic syndrome rat, independently of diuretic effect or blood pressure lowering effect. Thus, SGLT2 inhibition seems to be a promising therapeutic strategy for prediabetic metabolic syndrome.

**Electronic supplementary material:**

The online version of this article (doi:10.1186/s12933-016-0473-7) contains supplementary material, which is available to authorized users.

## Background

Type 2 diabetes is a major risk factor for cardiovascular disease [1, 2]. However, there is no convincing evidence that glucose-lowering therapy significantly reduces the rates of cardiovascular events and death, namely, macrovascular complications [3–9]. Sodium-glucose cotransporter 2 inhibitors [10, 11] are novel class of anti-hyperglycemic medications which increase urinary glucose excretion and thereby improving glycemic control independent of insulin. Recently, the EMPA-REG OUTCOME trial [12–14], which examined the effect of empagliflozin, a sodium glucose-cotransporter 2 inhibitor, in addition to standard therapy on cardiovascular morbidity and mortality in patients with type 2 diabetes at high risk, provided the findings that compared to placebo, empagliflozin significantly reduced the combined cardiovascular endpoint, cardiovascular death, overall mortality, and heart failure hospitalization. Thus, empagliflozin treatment shows the significant benefit in improving cardiovascular prognosis in high risk type 2 diabetic patients. However, the mechanism(s) underlying the reduction of cardiovascular events by empagliflozin reported in this trial remain to be elucidated. The potential non-glycemic benefit of SGLT2 inhibition has been paid the most attention since the reports of EMPA-REG OUTCOME trial.

Although SGLT2 inhibitors are shown to exert not only antihyperglycemic effect but also non-glycemic effects such as natriuresis, body weight reduction, blood pressure-lowering effect, or lipid-lowering effect, the previous reports regarding these non-glycemic effects of SGLT2 inhibitors are limited to diabetic animals [15–20] or patients [21–26]. The effect of SGLT2 inhibition on prediabetic metabolic syndrome is unknown. Therefore, to address this issue, in the present study, we investigated the effect of empagliflozin in a rat model of metabolic syndrome with prediabetic stage, focusing on its non-glycemic effects.

## Methods

### Ethics statement

All procedures were performed in accordance with the institutional guidelines for animal research and were approved by the Animal Care and Use Committee of Kumamoto University.

### Experimental animals

Male SHR/NDmcr-cp(+/+) rats (SHRcp) [27–29], a rat model of metabolic syndrome characterized by obesity, insulin resistance, impaired glucose tolerance with normal fasting blood glucose, hypertension, and hyperlipidemia, were purchased from Japan SLC (Shizuoka, Japan). The rats were housed in an animal facility with a 12 h light–dark cycle and were given water ad libitum.

### Drugs

Empagliflozin, a selective sodium-glucose cotransporter-2 (SGLT2) inhibitor, was kindly supplied by Boehringer Ingelheim Pharma GmbH & Co. (KG, Germany).

### Study protocol

The present study consisted of three experiments as described below.

#### Experiment I

This experiment was performed for metabolic cage analysis (see Additional file 1: Figure S1a). Twenty-week-old SHRcp were divided into two groups and were given (1) the standard diet (MF diet, ORIENTAL YEAST Co., Ltd, Tokyo, Japan) and (2) the standard diet containing 0.03% empagliflozin. Drug treatment was carried out for 10 weeks. During the first week (7 days), individual rats were housed in a metabolic cage (Techniplast 3701M001, Buguggiate, Italy) to collect 24-h urinary samples every day, and thereafter the animals were housed in a metabolic cage once a week for collection of 24-h urinary samples (see Additional file 1: Figure S1A). Urine volume, urinary excretions of glucose, Na and creatinine, food intake, and water intake were determined per 24 h in individual rats, and 24-h sodium or water balance, and cumulative 24-h sodium or water balance were calculated, as described below.

#### Experiment II

This experiment was performed to investigate the effect of long-term empagliflozin treatment on cardiac and fat tissues in SHRcp (see Additional file 1: Figure S1B). Twenty-two-week-old SHRcp rats were divided into two groups and were given (1) the standard diet or (2) the standard diet containing 0.03% empagliflozin for 10 weeks in the same manner as Experiment I. After 10 weeks of treatment, under anesthesia with isoflurane, blood was collected by cardiac puncture, and the heart, subcutaneous fat, epididymal fat, and liver were rapidly excised from each rat to examine the effects of empagliflozin on various parameters of cardiac tissue and fat tissue as described below. According to our previous report [27] on the detailed characteristics of SHRcp compared to control WKY, the slight difference in study age between Experiments I and II seems to be negligible.

#### Experiment III

This experiment was performed to continuously monitor the effect of empagliflozin on arterial blood pressure (BP) and heart rate (HR) of SHRcp, using radiotelemetry system (Data Science International, St Paul, MN, USA) (see Additional file 1: Figure S1C). The detail of our methods has been previously reported and the validity of our method has been well established [27, 30]. We used younger (12 to 13-week-old) SHRcp which has much less visceral fat than 20 to 22-week-old SHRcp, because older SHRcp has highly large amount of visceral fat tissue which hampers us to successfully implant telemetry device in older SHRcp. In brief, the tip of the transmitter catheter was inserted into the abdominal aorta of 12 to 13-week-old SHRcp and the transmitter was sutured to the ventral wall of the abdominal cavity. BP and HR were continuously recorded using a computer system (DATAQUEST ART4.2 Acquisition; Data Sciences International, St Paul, MN, USA). After recovery period, 15-week-old SHRcp were divided into two groups and were given (1) the standard diet or (2) the standard diet containing 0.03% empagliflozin for 7 weeks. The data were recorded with 30-s averages every 5 min for BP, HR and locomotor activity and with a 5-min average every 60 min. Spontaneous baroreceptor reflex gain (sBRG) was determined from spontaneous changes in systolic BP and pulse interval (PI) using a modified time-series method established by Oosting et al. and Waki et al. [30–33].

HR and systolic BP variability (low frequency (LF)/high frequency (HF) ratio of PI and LF of systolic BP) were calculated using data-analysis program contained a fast Fourier transform function for power spectral analysis established by Oosting et al. and Waki H et al. [30–33].

### Calculation of 24-h sodium balance, cumulative sodium balance, 24-h water balance, and cumulative water balance in Experiment I

In the above mentioned Experiment I, 24-h sodium intake was calculated by multiplying food intake (g) by the diet sodium content (0.19% NaCl = 0.082 mmol Na^+^/g food). Twenty-four-hour sodium excretion was calculated by multiplying the 24-h urine volume (mL) and urinary sodium concentration (mmol Na^+^/mL). Twenty-four-hour sodium balance was calculated as 24-h sodium intake minus 24-h sodium excretion. Twenty-four-hour water balance was calculated as 24-h water intake minus 24-h urine volume. Cumulative sodium or water balance was calculated from sequential summation of daily balances according to our previous report [34].

### Histological analysis and immunohistochemistry of the heart

To evaluate cardiac interstitial fibrosis, hearts were fixed with 4% formalin overnight, embedded in paraffin, cut into 4-μm thick coronal sections, and stained with Sirius Red F3BA (0.5% in saturated aqueous picric acid, Aldrich Chemical Company, Milwaukee, WI, USA). Positive fibrosis area per field area was assessed by examining at least 10 fields per rat using WinRoof Version 5.8 (Mitani Corporation, Fukui, Japan).

To evaluate cardiac interstitial macrophage infiltration, hearts were fixed with 4% formalin overnight, embedded in paraffin, cut into 4-μm thick coronal sections, and immunostained with anti-ED-1 antibodies (BMA Biomedicals, Augst, Switzerland) (working dilution 1:500) to identify monocytes/macrophages, as described previously [35]. The number of cardiac ED-1 positive cells per field area (mm^2^) was counted by examining more than ten fields per section using a microscope with 200× magnification; the average number of ED-1 positive cells was obtained for each rat.

To evaluate cardiomyocyte size [36], cardiac tissue were fixed with 4% formalin overnight, embedded in paraffin, cut into 4-μm thick coronal sections, and stained with fluorescein-tagged wheat germ agglutinin (FITC-WGA, Sigma-Aldrich, St. Louis, MO). Minimal Feret’s diameter of cardiomyocyte was assessed by examining at least 100 cell sizes per rat using WinRoof Version 5.8 (Mitani Corporation, Fukui).

### Measurement of cardiac superoxide

Dihydroethidium (DHE) was used to evaluate tissue superoxide levels in situ, as described previously [27]. DHE fluorescence of cardiac sections was quantified using WinRoof Version 5.8 (Mitani Corporation, Fukui). The mean fluorescence was quantified and expressed relative to values obtained from control rats.

### Measurement of triglyceride in left ventricular tissue

Triglyceride in left ventricular tissue was measured with a commercial assay kit (Bio Vision, Inc., CA, USA) according to the manufacturer’s recommended protocol.

### Measurement of adipocyte size and adiponectin in epididymal and subcutaneous adipose tissue

To evaluate adipocyte size, epididymal (visceral) and subcutaneous adipose tissue were fixed with 4% formalin overnight, embedded in paraffin, cut into 4-μm thick coronal sections, and stained with haematoxylin and eosin. Adipocyte size was assessed by examining at least 100 cell sizes per rat using WinRoof Version 5.8 (Mitani Corporation, Fukui).

Adiponectin protein levels in adipose tissues were analyzed using a mouse/rat adiponectin ELISA kit (Otsuka Pharmaceutical, Tokyo, Japan). Protein extraction from adipose tissue and protein quantification were performed as described by other investigators [37].

### Measurement of thiobarbituric acid reactive substrances (TBARS)

Lipid oxidation was evaluated by measuring the amounts of malondialdehyde (MDA) produced thiobarbituric acid reactive substances (TBARS), using a commercial assay kit (Cayman Chemical, Ann Arbor). Briefly, adipose tissue homogenate, sodium dodecyl sulphate, acetic acid and TBA were mixed. The mixtures were heated at 95 °C in a water bath for 60 min. After incubation the tubes were cooled to room temperature the upper organic layer was taken and its OD read at 532 nm against an appropriate blank without the sample.

### Measurement of urinary and blood variables

Serum total cholesterol, triglyceride, and free fatty acid, and urinary electrolytes were measured at SRL, Inc. (Tokyo, Japan). Measurement of HbA1C was performed at biopathological medicine (Kanagawa, Japan). Plasma insulin was measured with a kit (Morinaga Institute of Biological Science, Inc, Yokohama).

### Statistical analysis

All of the data are presented as mean ± SEM. The difference among comparison groups was tested with appropriate statistical method, as shown in each Figure legend and tables. P < 0.05 was considered significant. Statistical analyses were performed using GraphPad Prism version 6.0 for Windows (GraphPad Software, San Diego, CA).

## Results

### The effects of short-term (7 days) empagliflozin treatment on daily body weight, food intake, water intake, urine volume, urinary glucose and sodium excretions, and water and sodium balances in SHRcp

**Fig. 1:**
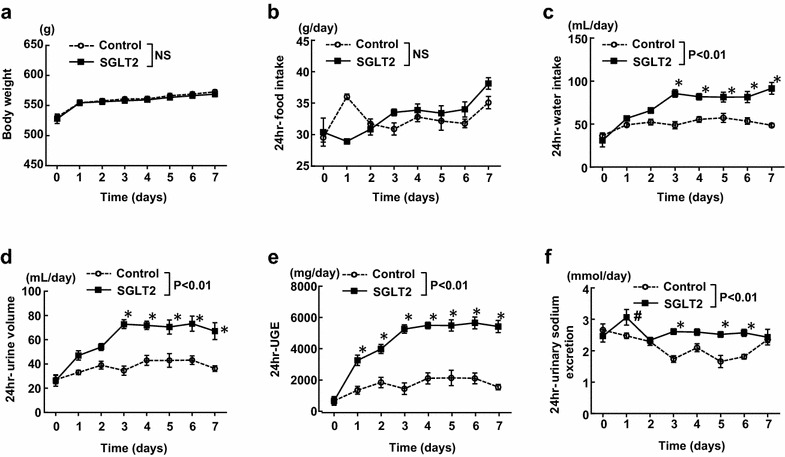
Effect of short-term (7 days) empagliflozin treatment on various parameters (**a**–**f**) obtained from daily metabolic cage analysis of SHRcp. *Control* SHRcp fed control diet; *SGLT2*, SHRcp fed control diet containing empagliflozin; *UGE* urinary glucose excretion; *NS* not significant. Values are mean ± SEM n = 6 in control, n = 5 in SGLT2. Statistical analysis was performed by two-factor ANOVA with repeated measures followed by post hoc Bonferroni’s multiple comparisons test. ^#^P < 0.05, *P < 0.01 versus control

**Fig. 2:**
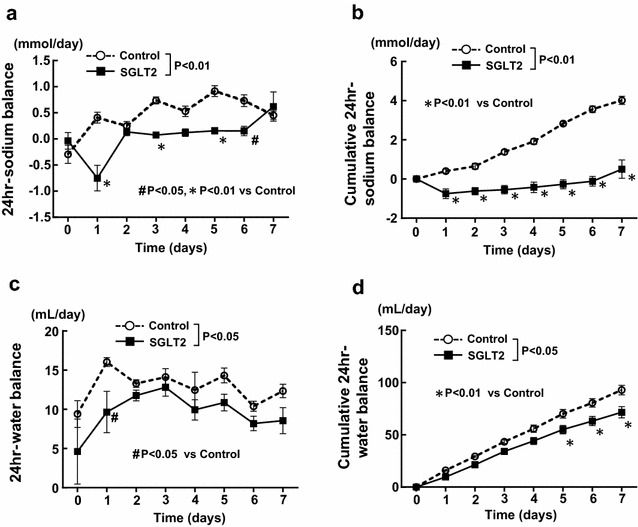
Effect of short-term (7 days) empagliflozin treatment on daily 24-h sodium balance (**a**), cumulative 24-h sodium balance (**b**), 24-h water balance (**c**), and cumulative 24-h water balance (**d**) of SHRcp. *Control* SHRcp fed control diet; *SGLT2*, SHRcp fed control diet containing empagliflozin; *UGE* urinary glucose excretion; *NS* not significant. Values are mean ± SEM n = 6 in control, n = 5 in SGLT2. Statistical analysis was performed by two-factor ANOVA with repeated measures followed by post hoc Bonferroni’s multiple comparisons test. ^#^P < 0.05, *P < 0.01 versus control

There was no significant difference in daily body weight and daily food intake between control and empagliflozin groups during 7 days of the treatment (Fig. 1a, b), but there was a significant interaction (P < 0.01) regarding daily food intake (Table 1). However, compared with control group, empagliflozin significantly increased daily water intake, urine volume, and urinary glucose excretion in SHRcp (Fig. 1c–e). Furthermore, 24-h urinary sodium excretions were larger in empagliflozin group than those in control group (Fig. 1f), and there was a significant interaction (P < 0.01) regarding 24-h urinary sodium excretion (Table 1). As shown in Fig. 2, relative to control, empagliflozin significantly decreased daily sodium balance and cumulative 24-h sodium balance, and significantly reduced cumulative 24-h water balance within 7 days of the treatment.

**Table 1:** Main effects and interaction P values for drug and time analyzed by two-way ANOVA in Experiment I

Parameters (figure number)	*P*_*time*_	*P*_*drug*_	*P*_*interaction*_
Body weight (Fig. 1a)	<0.01	NS	NS
24 h-food intake (Fig. 1b)	<0.01	NS	<0.01
24 h-water intake (Fig. 1c)	<0.01	<0.01	<0.01
24 h-urine volume (Fig. 1d)	<0.01	<0.01	<0.01
24 h-UGE (Fig. 1e)	<0.01	<0.01	<0.01
24 h-urinary sodium excretion (Fig. 1f)	<0.01	<0.01	<0.01
24 h-sodium balance (Fig. 2a)	<0.01	<0.01	<0.01
Cumulative 24 h-sodium balance (Fig. 2b)	<0.01	<0.01	<0.01
24 h-water balance (Fig. 2c)	<0.01	<0.05	0.6951
Cumulative 24 h-water balance (Fig. 2d)	<0.01	<0.05	<0.01
Body weight (Fig. 3a)	<0.01	<0.05	<0.01
24 h-food intake (Fig. 3b)	<0.01	<0.01	<0.01
24 h-water intake (Fig. 3c)	<0.01	<0.01	<0.01
24 h-urine volume (Fig. 3d)	<0.01	<0.01	<0.01
24 h-UGE (Fig. 3e)	<0.01	<0.01	<0.01
24 h-urinary sodium excretion (Fig. 3f)	<0.01	NS	<0.01
24 h-sodium balance (Fig. 3g)	<0.01	NS	NS
24 h-water balance (Fig. 3h)	<0.01	NS	<0.01

*UGE* urinary glucose excretion; *NS* not significant

### The effect of long-term (10 weeks) empagliflozin treatment on body weight, food intake, water intake, urine volume, urinary glucose and sodium excretions, and water and sodium balances in SHRcp

**Fig. 3:**
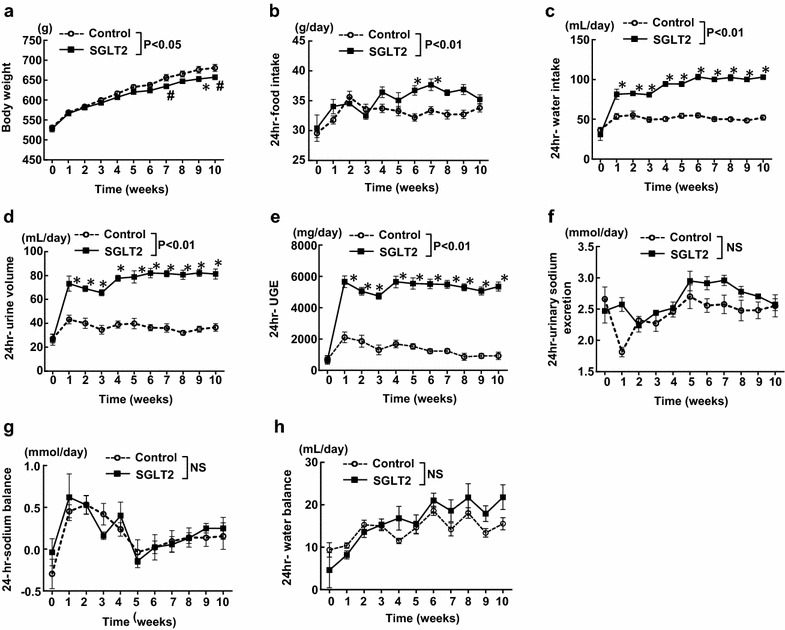
Effect of long-term (10 weeks) empagliflozin treatment on various parameters (**a**–**h**) obtained from weekly metabolic cage analysis. *Control* SHRcp fed control diet; *SGLT2*, SHRcp fed control diet containing empagliflozin; *UGE* urinary glucose excretion; *NS* not significant. Values are mean ± SEM n = 6 in control, n = 6 in SGLT2. Statistical analysis was performed by two-factor ANOVA with repeated measures followed by post hoc Bonferroni’s multiple comparisons test

Body weight began to be significantly less in empagliflozin group than in control group from 7 weeks after initiation of empagliflozin treatment (Fig. 3a), while 24-h food intake began to be significantly greater in empagliflozin group than in control group from 5 weeks (Fig. 3b). As with the above mentioned short-term effects of empagliflozin treatment, 24-h water intake, urine volume, and urinary glucose excretions continued to be greater in empagliflozin group than in control group from 1 week until 10 weeks of the treatment (Fig. 3c, d, e).

Different from the shot-term (7 days) effects of empagliflozin, there was no significant difference in 24-h urinary sodium excretion, sodium balance, and water balance between empagliflozin and control group until the end of the treatment (10 weeks) (Fig. 3f, g, h). However, there was a significant interaction (P < 0.01) regarding 24-h water balance (Table 1).

### The effects of empagliflozin treatment on organ weight in SHRcp

After 10 weeks of the treatment, body weight was significantly less in empagliflozin group versus control group (664.2 ± 9.2 versus 733.3 ± 5.3 g; P < 0.01), while tibia length was similar between the groups (Table 2). There was no significant difference between the empagliflozin and control groups regarding the weight of visceral fat tissues including epididymal fat (7.0 ± 0.3 versus 6.4 ± 0.2 g), mesenteric fat (11.8 ± 0.2 versus 12.3 ± 0.2 g), and perirenal fat (53.8 ± 1.3 versus 56.2 ± 1.0 g) (Table 2). On the other hand, subcutaneous fat weight was significantly less in empagliflozin group versus control group (52.7 ± 2.2 versus 69.0 ± 1.5 g; P < 0.01), (Table 2). There was no significant difference in liver weight (Table 2).

**Table 2:** Effect of long-term (10 weeks) empagliflozin treatment on body weight, tibia length, visceral fat weight, subcutaneous fat weight, liver weight, and left ventricular weight of SHRcp

	Control (n = 12)	SGLT2 (n = 12)
Body weight (g)	733.3 ± 5.3	664.0 ± 9.2*
Tibia length (mm)	39.1 ± 0.1	39.0 ± 0.1
Epididymal fat weight (g)	6.4 ± 0.2	7.0 ± 0.3
Mesenteric fat weight (g)	12.3 ± 0.2	11.8 ± 0.2
Perirenal fat weight (g)	56.2 ± 1.0	53.8 ± 1.3
Subcutaneous fat weight (g)	69.0 ± 1.5	52.7 ± 2.2*
Liver weight (g)	29.5 ± 0.74	28.6 ± 0.35
Left ventricular weight (mg)	1465 ± 44	1363 ± 16^#^

*Control* SHRcp fed control diet; *SGLT2* SHRcp fed control diet containing empagliflozin. Values are mean ± SEM. Statistical analysis was performed by unpaired Student’s t test

^#^ P < 0.05, * P < 0.01 versus control

Left ventricular weight was significantly less in empagliflozin group versus control group (1363 ± 16 versus 1465 ± 44 mg; P < 0.05) (Table 2).

### The effects of empagliflozin treatment on adipocyte size distribution in visceral and subcutaneous adipose tissues

**Fig. 4:**
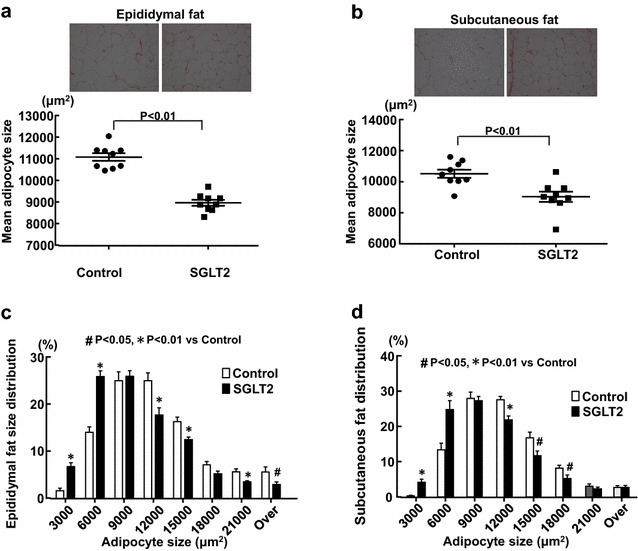
Effect of long-term (10 weeks) empagliflozin treatment on epididymal adipocyte size (**a**, **c**) and subcutaneous adipocyte size (**b**, **d**) of SHRcp. *Control* SHRcp fed control diet; *SGLT2*, SHRcp fed control diet containing empagliflozin; *UGE* urinary glucose excretion; *NS* not significant. Values are mean ± SEM n = 9 in control, n = 9 in SGLT2. In **a** and **b**, statistical analysis was performed by unpaired Student’s t test. In **c** and **d**, statistical analysis was performed by two-factor ANOVA with repeated measures followed by post hoc uncorrected Fisher’s LSD multiple comparisons test

Figure 4 indicates morphological analysis of epididymal (visceral) fat and subcutaneous fat tissues. In both epididymal (P < 0.01) and subcutaneous (P < 0.01) fat tissues, mean adipocyte size in empagliflozin group was significantly smaller than that in control group (Fig. 4a, b, respectively). Furthermore, in both epididymal and subcutaneous fat tissue, empagliflozin group had higher proportion of small size of adipocytes and lower proportion of large size of adipocytes than control group (Fig. 4c, d, respectively).

### Effect of empagliflozin treatment on adiponectin and oxidative stress of adipose tissue

There is no significant difference between the control and empagliflozin groups regarding epididymal fat adiponectin (1.16 ± 0.07 versus 1.03 ± 0.08 ng/μg protein) and subcutaneous fat adiponectin (1.42 ± 0.09 versus 1.37 ± 0.08 ng/μg protein).

**Fig. 5:**
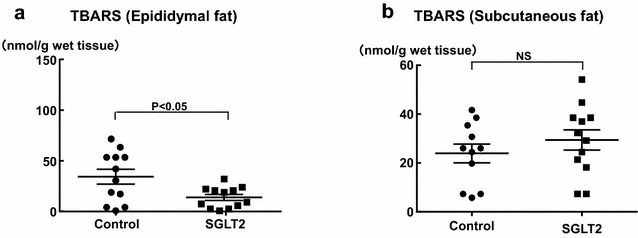
Effect of long-term (10 weeks) empagliflozin treatment on epididymal (**a**) and subcutaneous (**b**) fat tissue TBARS of SHRcp. *Control* SHRcp fed control diet; *SGLT2*, SHRcp fed control diet containing empagliflozin; *UGE* urinary glucose excretion; *NS* not significant. Values are mean ± SEM n = 11–12 in control, n = 12 in SGLT2. Statistical analysis was performed by unpaired Student’s t test

As shown in Fig. 5, epididymal fat TBARS (lipid oxidation) levels were less in empagliflozin group than in control group (P < 0.05), while there was no significant difference between the two groups regarding subcutaneous fat TBARS.

### Effect of empagliflozin treatment on cardiac injury of SHRcp

**Fig. 6:**
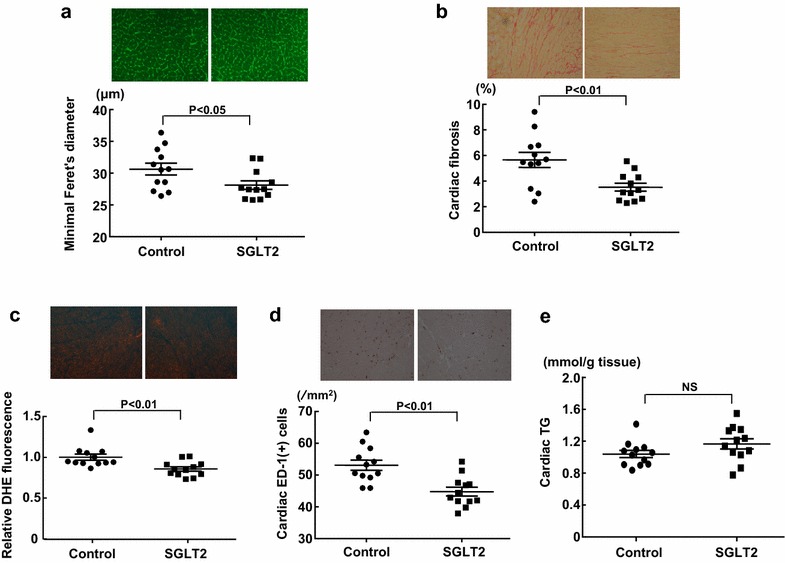
Effect of long-term (10 weeks) empagliflozin treatment on cardiomyocyte minimal Feret’s diameter (**a**), cardiac interstitial fibrosis (**b**), cardiac DHE fluorescence (**c**), cardiac ED-1-positive cell numbers (**d**), and cardiac triglyceride contents (**e**) of SHRcp. *Control* SHRcp fed control diet; *SGLT2*, SHRcp fed control diet containing empagliflozin; *UGE* urinary glucose excretion; *NS* not significant. Values are mean ± SEM n = 12 in control, n = 12 in SGLT2. Statistical analysis was performed by unpaired Student’s t test

As described above (Table 2), left ventricular weight was significantly less in empagliflozin group than in control group. Therefore, we performed histological analysis of cardiac tissue in SHRcp. As shown in Fig. 6a, empagliflozin significantly reduced minimal Feret’s diameter of cardiomyocyte in SHRcp (P < 0.05). Moreover, empagliflozin ameliorated cardiac interstitial fibrosis (P < 0.01), superoxide levels (P < 0.01), and ED-1-positive cell infiltration (P < 0.01) in SHRcp (Fig. 6b, c, d). Cardiac triglyceride levels were not significantly different between the two groups (Fig. 6e).

### The effects of empagliflozin treatment on serum biochemical parameters in SHRcp

As shown in Table 3, after 10 weeks of the treatment, empagliflozin significantly decreased non-fasting blood glucose (P < 0.01), HbA1c (P < 0.01) and serum insulin (P < 0.01) than control, but did not change serum total cholesterol and free fatty acid, and increased triglyceride levels (P < 0.01) than control.

**Table 3:** Non-fasting blood sugar, HbA1c, insulin, and lipids in SHRcp fed empagliflozin-containing diet or control diet for 10 weeks

	Control (n = 11–12)	SGLT2 (n = 12)
Non-fasting blood sugar (mg/dl)	227 ± 17	140 ± 6*
HbA1c (%)	1.30 ± 0.03	1.12 ± 0.02*
Insulin (ng/mL)	103.6 ± 12.2	22.4 ± 2.4*
Total cholesterol (mg/dL)	206 ± 6	207 ± 9
Triglyceride (mg/dL)	591 ± 51	812 ± 39*
Free fatty acid (mmol/L)	0.46 ± 0.03	0.48 ± 0.02

*Control* SHRcp fed control diet; *SGLT2* SHRcp fed control diet containing empagliflozin. Values are mean ± SEM. Statistical analysis was performed by unpaired Student’s t test

* P < 0.01 versus control

### Effects of empagliflozin treatment on blood pressure, heart rate, and locomotor activity in SHRcp

**Fig. 7:**
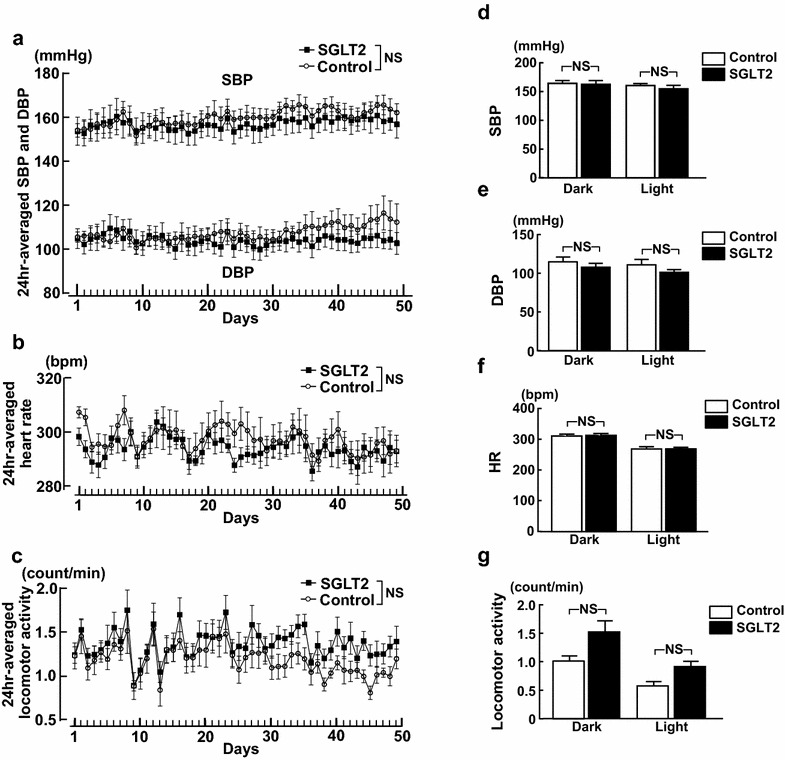
Effect of 7 weeks of empagliflozin treatment of systolic BP and diastolic BP (**a**), heart rate (**b**), locomotor activity (**c**), and circadian rhythm during 24 h (12-h dark period and 12-h light period) of systolic BP (**d**), diastolic BP (**e**), heart rate (**f**), and locomotor activity (**g**) of SHRcp as estimated by telemetry system. *Control* SHRcp fed control diet; *SGLT2*, SHRcp fed control diet containing empagliflozin; *UGE* urinary glucose excretion; *NS* not significant. *SBP* systolic blood pressure; *DBP* diastolic blood pressure; *HR* heart rate. Values are mean ± SEM n = 5 in control, n = 6 in SGLT2. In **a**, **b** and **c**, statistical analysis was performed by two-factor ANOVA with repeated measures. In **d**, **e**, **f** and **g**, statistical analysis was performed by two-factor ANOVA followed by post hoc Tukey’s multiple comparisons test

Blood pressure and heart rate were continuously monitored in SHRcp for 7 weeks by using telemetry system. As shown in Fig. 7, there was no significant difference between empagliflozin and control groups throughout the treatment, regarding 24-h-averaged systolic BP and diastolic BP (Fig. 7a), 24-h-averaged HR (Fig. 7b), or 24-h-averaged locomotor activity (Fig. 7c). Furthermore, as shown by circadian rhythm hourly recorded during 24 h (12-h dark period and 12-h light period) in Fig. 7d–g, there was no significant difference between empagliflozin and control groups regarding systolic BP, diastolic BP, HR, or locomotor activity during 12-h dark period and 12-h light period (Table 4).

**Table 4:** Main effects and interaction P values for drug and time analyzed by two-way ANOVA in Experiment III (experiment using radiotelemetry system)

Parameters (figure number)	*P*_*time*_	*P*_*drug*_	*P*_*interaction*_
24 h-averaged SBP (Fig. 7a)	<0.01	NS	NS
24 h-averaged DBP (Fig. 7a)	<0.01	NS	<0.01
24 h-averaged heart rate (Fig. 7b)	<0.01	NS	NS
24 h-averaged locomotor activity (Fig. 7c)	<0.01	NS	NS
Hourly SBP (Fig. 7d)	<0.01	NS	NS
Hourly DBP (Fig. 7e)	<0.01	NS	NS
Hourly heart rate (Fig. 7f)	<0.01	NS	NS
Hourly locomotor activity (Fig. 7g)	<0.01	P < 0.05	NS
LF-SBP (Fig. 8a)	<0.01	NS	NS
sBRG (Fig. 8b)	<0.01	NS	NS
LF/HF ratio of PI (Fig. 8c)	<0.01	NS	NS

*SBP* systolic blood pressure; *DBP* diastolic blood pressure; *LF* low frequency; *sBRG* spontaneous baroreceptor reflex gain; *HF* high frequency; *PI* pulse interval; *NS* not significant

### Effects of empagliflozin treatment on autonomic function in SHRcp

**Fig. 8:**
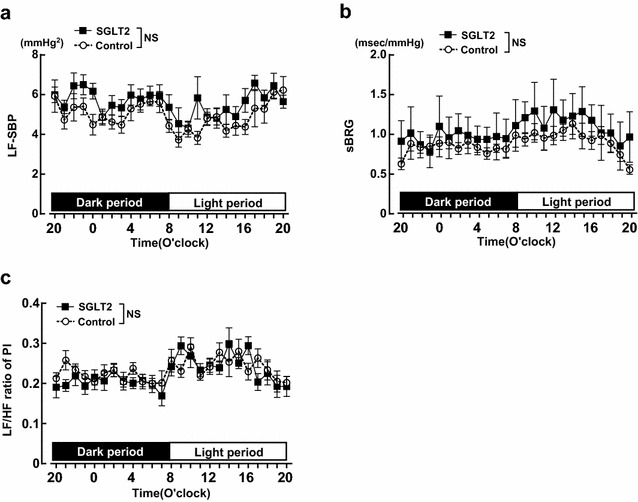
Effect of 7 weeks of empagliflozin treatment on circadian rhythm of LF-SBP (**a**), sBRG (**b**), and LF/HF ratio of PI (**c**) during 24 h (12-h dark period and 12-h light period) of SHRcp as estimated by telemetry system. *Control* SHRcp fed control diet; *SGLT2*, SHRcp fed control diet containing empagliflozin; *UGE* urinary glucose excretion; *NS* not significant. *PI* pulse interval. Values are mean ± SEM n = 5 in control, n = 6 in SGLT2. Statistical analysis was performed by two-factor ANOVA with repeated measures

Figure 8 indicates hourly LF-SBP, sBRG, and LF/HF ratio of PI in each group of SHRcp rats on 45 day after start of drug treatment. Compared with control, empagliflozin treatment did not alter LF-SBP, sBRG, and LF/HF ratio of PI during 24 h (12-h dark period and 12-h light period) in SHRcp.

## Discussion

At present, available data regarding the effect of SGLT2 inhibition on MS with prediabetic stage is very scarce. The major findings of our present work were as follows: (1) empagliflozin significantly ameliorated cardiac hypertrophy and interstitial fibrosis in association with the attenuation of cardiac oxidative stress and inflammation in prediabetic MS rat, independently of blood pressure and lipid metabolism; (2) empagliflozin reduced visceral adipocyte hypertrophy in prediabetic MS rats. These results provided the first evidence suggesting that SGLT2 inhibition with empagliflozin may provide the therapeutic benefit in MS with prediabetic stage.

Recently, EMPA-REG OUTCOME trial provides the evidence that SGLT2 inhibition with empagliflozin significantly reduces cardiovascular events and heart failure hospitalization in type 2 diabetic patients at high risk [12–14, 38]. However, the underlying mechanism for the benefit of empagliflozin in cardiovascular risk reduction remains to be determined. Interestingly, SGLT2 inhibitors including empagliflozin exert non-glycemic effect in type 2 diabetic patients [11, 21, 24–26] or animal models [16, 17, 19, 39], such as reduction in blood pressure, decrease in body weight, or natriuresis. Therefore, SGLT2 inhibition is proposed to provide cardiovascular protection partially independently of blood glucose control. However, the investigation on the effect of SGLT2 inhibition is limited to type 2 diabetic humans and animals, and its effect on prediabetic MS is unknown. These findings encouraged us to investigate the effect of empagliflozin on prediabetic MS rat model. In our previous paper, we have characterized in detail the phenotype of SHRcp compared to genetic control Wistar-Kyoto rats (WKY) [27]. We showed that compared to control WKY rat, SHRcp exhibited visceral obesity, insulin resistance, impaired glucose tolerance with fasting normoglycemia, hypertension, and hyperlipidemia, thereby indicating that SHRcp is a useful model of MS with prediabetic stage [27].

Clinical studies showed that empagliflozin monotherapy reduced postprandial glucose from the first day and improved 24-h glucose variability in Japanese patients with type 2 diabetes [40], the combination of empagliflozin with metformin significantly reduced HbA1c with good tolerability in type 2 diabetic patients [41], and long-term empagliflozin monotherapy in drug-naive patients with type 2 diabetes led to sustained reductions in HbA1c and weight compared to placebo [42]. In the present study, according to the method of our previous report [39], empagliflozin was administered in the diet (containing 0.03% empagliflozin), because the diet containing such a concentration of empagliflozin is shown to be suitable for investigating the long-term effect of empagliflozin in diabetic animals. As expected, empagliflozin significantly increased urinary glucose excretion in prediabetic MS rats throughout 10 weeks of the treatment, being associated with the slight but significant reduction in HbA1c and the significant decrease in plasma insulin levels. The reduction of plasma insulin levels by empagliflozin in SHRcp seems to be secondary to the normalization of blood glucose levels but not the direct effect on insulin secretion. Furthermore, greater urinary sodium excretion, smaller sodium balance, and smaller water balance in empagliflozin than in control were observed within the initial one week of the treatment, thereby indicating the significant natriuresis by empagliflozin in prediabetic SHRcp. On the other hand, later than one week of the treatment, urinary sodium excretion and urinary sodium or water balance were similar between empagliflozin and control groups of SHRcp. The lack of difference between the groups in urinary sodium or water balance during prolonged treatments is likely to be accounted for by the action of other renal Na transporters, although further study is required to elucidate this point.

### Effects of empagliflozin on blood pressure

Convincing evidence [11, 21, 26] indicates that SGLT2 inhibitors including empagliflozin significantly reduce BP in type 2 diabetic patients and natriuretic action of SGLT2 inhibitors is postulated to participate in BP reduction. However, there is no available information concerning the effect of SGLT2 inhibition on BP in prediabetic MS. In our previous paper, we have shown that SHRcp exhibits higher blood pressure and the increased sympathetic activity compared to control WKY rats [27]. Therefore, SHRcp is a suitable model to investigate the effect of empagliflozin on blood pressure or sympathetic activity in prediabetic MS rats. In the present study, by using radiotelemetry system, we continuously monitored the long-term effect of empagliflozin on direct BP, HR, baroreflex function, and autonomic activity in prediabetic MS rats. Our continuous direct monitoring indicated that empagliflozin treatment did not significantly alter systolic or diastolic BP and HR or their circadian rhythm in SHRcp. Furthermore, empagliflozin did not affect autonomic nerve activity or baroreceptor function in SHRcp, as evidenced by no alteration of LF-SBP, sBRG, or LF/HF ration of PI. Our results on BP of prediabetic MS rats differ from clinical findings observed in type 2 diabetic patients. However, it should be noted that in the present study, SHRcp were fed normal salt diet but not high-salt diet. Furthermore, osmotic diuresis caused by glycosuria appears to be much less in prediabetic SHRcp than in diabetic animals, because SHRcp exhibited much less blood glucose levels than diabetic animal models, and therefore, the extent of glycosuria induced by empagliflozin in SHRcp was slight.

### Effects of empagliflozin on cardiac injury

The potential effect of SGLT2 inhibition on cardiac disease is paid the most attention, since empagliflozin is demonstrated to reduce cardiovascular events and heart failure hospitalization in type 2 diabetic patients [12–14, 38]. To address this issue, we investigated the effect of empagliflozin on cardiac injury in SHRcp, since we have previously shown that SHRcp displays cardiac hypertrophy, cardiac fibrosis, cardiac inflammation, and cardiac oxidative stress more than control WKY rat [27]. In the present study, of note, empagliflozin significantly reduced cardiac myocyte hypertrophy and interstitial fibrosis in SHRcp, despite no significant lowering of BP by empagliflozin. Furthermore, empagliflozin significantly reduced cardiac oxidative stress and inflammation but did not change cardiac triglyceride contents and did not reduce plasma lipids in SHRcp. Taken together with the fact that oxidative stress and inflammation play a pivotal role in the pathogenesis of cardiac hypertrophy and remodeling [43–45], our present observations show that the amelioration of cardiac hypertrophy and fibrosis by empagliflozin in SHRcp was attributed to the attenuation of oxidative stress and inflammation but not to BP. However, at present, cardioprotection of empagliflozin observed in this work seems unlikely to be mediated by its direct cardiac action, because SGLT2 receptor does not exist in cardiac tissue. Further study is required to elucidate the precise mechanism responsible for cardioprotection by empagliflozin in prediabetic MS.

### Effects of empagliflozin on obesity

Accumulating studies provide the solid evidence that long-term SGLT2 inhibition causes the reduction of body weight in type 2 diabetic patients [11, 24]. However, the underlying mechanism remains to be clarified. Therefore, in the present study, we examined the effect of empagliflozin on body weight and adipose tissues in SHRcp. We found that long-term SGLT2 inhibition significantly decreased body weight in prediabetic MS rats and significantly reduced subcutaneous fat tissue weight but not visceral fat tissue weight. Therefore, the decrease in body weight of prediabetic MS rats subjected to empagliflozin treatment seems to be attributed to the decrease in subcutaneous fat tissue rather than visceral fat. However, of note, empagliflozin treatment significantly increased the proportion of smaller adipocytes while decreased the proportion of larger adipocyte in visceral (epididymal) adipose tissue, indicating the amelioration of visceral adipocyte hypertrophy by empagliflozin. Moreover, this amelioration of visceral adipocyte hypertrophy by empagliflozin was accompanied by the reduction of oxidative stress (lipid oxidation). Our present observations provided the first evidence that SGLT2 inhibition with empagliflozin caused the reduction in visceral adipocyte hypertrophy and the reduction in oxidative stress in prediabetic MS rats. On the other hand, unlike visceral (epididymal) fat, subcutaneous fat oxidative stress was not altered by empagliflozin. Thus, the effect of empagliflozin seems to be different between visceral and subcutaneous fat tissues. Furthermore, unexpectedly, serum triglyceride levels were higher in empagliflozin group than in control group. The increased triglyceride levels in empagliflozin group may be attributed to the increased food intake in this group, although further study is required to elucidate this point.

In conclusion, we first examined the detailed effect of SGLT2 inhibition on prediabetic MS rats. We obtained the evidence that empagliflozin ameliorated cardiac hypertrophy and fibrosis in prediabetic MS rats, through attenuation of oxidative stress and inflammation, independently of BP. Furthermore, empagliflozin treatment provided the reduction in visceral adipocyte hypertrophy and the attenuation of visceral adipocyte oxidative stress in prediabetic MS rats. Thus, our present work highlights SGLT2 inhibition as a novel therapeutic strategy for MS with prediabetic stage.

## Additional file

**Additional file 1: Figure S1.** Protocol of Experiment I (A), Experiment II (B), and Experiment III (C).

## Abbreviations

BP: blood pressure
LF: low frequency
HR: heart rate
sBRG: spontaneous baroreceptor reflex function
SGLT-2: sodium glucose-cotransporter 2
PI: pulse interval
