# Supplementary material for: Empagliflozin lessened cardiac injury and reduced visceral adipocyte hypertrophy in prediabetic rats with metabolic syndrome
Source: Cardiovasc Diabetol. 2016 Nov 11;15:157. doi: 10.1186/s12933-016-0473-7 (PMC5106779; doi:10.1186/s12933-016-0473-7)
Supplement: Supplementary file 1 — Additional file 1: Figure S1. Protocol of Experiment I (A), Experiment II (B), and Experiment III (C). [file 12933_2016_473_MOESM1_ESM.docx]

**
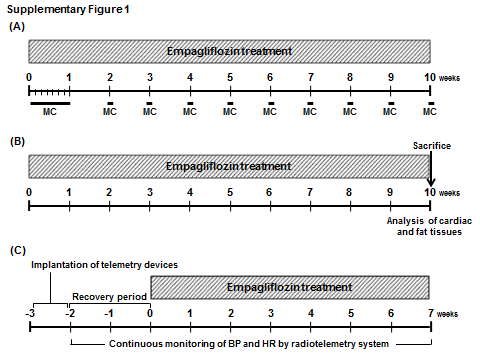
**

**Supplementary Figure 1.** Protocol of Experiment I (A), Experiment II (B), and Experiment III (C)

In (A), MC indicates housing of individual rats in metabolic cage for collection of urine samples. During the initial 7 days, individual rats were housed in metabolic cage to collect 24-hour urinary samples every day. From the 2nd week, individual rats were housed in metabolic cage once a week to collect 24-hour urinary samples.

Abbreviations used in (C): BP, blood pressure; HR, heart rate.
